# Role of Circular RNAs in Atherosclerosis through Regulation of Inflammation, Cell Proliferation, Migration, and Apoptosis: Focus on Atherosclerotic Cerebrovascular Disease

**DOI:** 10.3390/medicina59081461

**Published:** 2023-08-14

**Authors:** Zheng Zhang, Lingfei Li, Huanqing Shi, Biao Chen, Xiaoqin Li, Yuyao Zhang, Fei Liu, Wan Wei, Yongji Zhou, Keqin Liu, Wenqing Xia, Xin Gu, Jinyu Huang, Sheng Tu, Congguo Yin, Anwen Shao, Lin Jiang

**Affiliations:** 1 The Fourth School of Clinical Medicine, Zhejiang Chinese Medical University, Hangzhou 310053, China; zzneurology@163.com (Z.Z.); 17698573086@139.com (H.S.); cb1105156722@163.com (B.C.); lxq1875822@163.com (X.L.); wo2010zyy@163.com (Y.Z.); wmuguxin@163.com (X.G.); 2 Department of Neurology, Affiliated Hangzhou First People’s Hospital, Zhejiang University School of Medicine, Hangzhou 310003, China; leelingfei@126.com (L.L.); liufei@mail.ccmu.edu.cn (F.L.); weiwan18757143281@163.com (W.W.); jessie52014@163.com (Y.Z.); liukeqin@zju.edu.cn (K.L.); xiawenqing06170604@126.com (W.X.); 3 Department of Cardiology, Affiliated Hangzhou First People’s Hospital, Zhejiang University School of Medicine, Hangzhou 310006, China; zdsyhjy0902@zju.edu.cn; 4 State Key Laboratory for Diagnosis and Treatment of Infectious Diseases, Collaborative Innovation Center for Diagnosis and Treatment of Infectious Diseases, The First Affiliated Hospital, College of Medicine, Zhejiang University, Hangzhou 310006, China; tusheng118@163.com; 5 Department of Neurosurgery, The Second Affiliated Hospital, School of Medicine, Zhejiang University, Hangzhou 310009, China; 6 Key Laboratory of Precise Treatment and Clinical Translational Research of Neurological Disease, Hangzhou 310009, China

**Keywords:** atherosclerosis, circular RNA (circRNA), inflammation, cell apoptosis, cell proliferation, cell migration

## Abstract

Atherosclerosis (AS) is a disease dangerous to human health and the main pathological cause of ischemic cardiovascular diseases. Although its pathogenesis is not fully understood, numerous basic and clinical studies have shown that AS is a chronic inflammatory disease existing in all stages of atherogenesis. It may be a common link or pathway in the pathogenesis of multiple atherogenic factors. Inflammation is associated with AS complications, such as plaque rupture and ischemic cerebral infarction. In addition to inflammation, apoptosis plays an important role in AS. Apoptosis is a type of programmed cell death, and different apoptotic cells have different or even opposite roles in the process of AS. Unlike linear RNA, circular RNA (circRNA) a covalently closed circular non-coding RNA, is stable and can sponge miRNA, which can affect the stages of AS by regulating downstream pathways. Ultimately, circRNAs play very important roles in AS by regulating inflammation, apoptosis, and some other mechanisms. The study of circular RNAs can provide new ideas for the prediction, prevention, and treatment of AS.

## 1. Introduction

Atherosclerosis (AS) is a chronic systemic inflammatory disease affecting large and medium-sized arteries that is thought to be an important cause of cardiovascular and cerebrovascular disease [1,2,3]. Worldwide, stroke and coronary heart disease (CHD) are the primary fatal and disabling diseases, and they pose a great burden to society [4,5]. AS is characterized by the accumulation of oxidized low-density lipoprotein (ox-LDL), increased inflammatory cytokines in the vessel wall, endothelial and vascular smooth muscle cell dysfunction, and monocyte/macrophage-initiated foam cell formation [6,7,8,9]. Currently, AS treatment focuses on preventing plaque growth and instability and reducing the risk of plaque rupture. Studies have shown that the lower the LDL cholesterol (LDL-C) level is, the more plaque progression can be delayed or even reversed [10]. Statins target hepatocytes through inhibiting 3-hydroxy-3-methylglutaryl-coenzyme A reductase (HMG-CoA), a key regulator of cholesterol biosynthesis [11]. Statins have been shown to significantly reduce the incidence of adverse events in coronary heart disease [10]. However, statins can be hepatotoxic and cause abnormal liver function [12,13,14]. PCSK9 inhibitors have been shown to reduce plasma LDL-C levels by approximately 60%, significantly reducing the incidence of macrovascular events [15]. However, the high cost of PCSK9 inhibitors may hinder long-term patient adherence to this treatment [16]. Therefore, further research focused on the pathogenesis and treatment of AS is needed.

Characterized by their unique structure, circular RNAs (circRNAs) are a class of covalently closed, circular non-coding RNAs generated from the reverse splicing of messenger RNA precursors (pre-messenger RNAs) [17,18]. CircRNAs are mostly formed by exon cyclization and shearing [19] and can be grouped into five types depending on where they are located in the genome: a) circular RNA genome; b) circular RNA intron; c) circular RNA processing intermediate; d) circular noncoding RNA; and e) circular RNA spliced exons [20]. MicroRNAs (miRNAs) are important post-transcriptional regulators of gene expression that function by direct base pairing with target sites in the untranslated area of messenger RNA (mRNA) [21]. The regulatory role of miRNAs is mainly through binding to the 3′ non-transcriptional regions (UTRs), 5′ UTRs, or coding regions of mRNAs, resulting in mRNA destabilization or translational repression [22,23]. MiRNA function may be regulated post-transcriptionally through competition between target mRNAs and non-target mRNAs (i.e., sponges) [24]. These RNAs are a competitive endogenous RNA (ceRNA) [24]. CeRNA can interact with miRNA response elements [25]. Studies have shown that there are multiple binding sites between circRNAs and corresponding miRNAs just like ceRNAs, and the combination of the two can affect the transcriptional regulation of miRNAs, which is the “sponge effect” of circRNAs [26]. After miRNAs interact with target mRNAs, miRNAs are degraded, whereas binding to sponge reduces miRNA activity but still maintains miRNA stability [27,28,29,30]. It follows that circRNAs affect the transcriptional expression of the corresponding mRNAs by influencing the activity of miRNAs. An increasing number of recent studies have shown that circRNAs play key roles in a variety of biological processes, including gene regulation, cell proliferation and differentiation, and disease genesis and development [31,32]. Among these processes, the relationship between circRNAs and AS has received much attention. The expression levels of different circRNAs appear to be upregulated or downregulated in the serum of AS patients [33,34]. Currently, available evidence suggests that circRNAs can affect inflammation and cell proliferation, migration, and apoptosis in AS patients or ox-LDL treated cells through multiple pathways. Because they can affect the development of AS, circRNAs may thus have predictive and therapeutic value, which could compensate for the shortcomings of existing therapeutic regimens.

As evidenced by the extremely high disease burden to human society caused by AS [4,5,35], the available diagnostic and treatment tools are somewhat inadequate; available evidence proves that circRNA has certain therapeutic effects on AS, and this review describes the specific role of circRNAs in AS in recent years.

## 2. The Role of CircRNAs in the Pathogenesis of AS

### 2.1. Endothelial Dysfunction

The first sign of AS is fatty streaks, and their initial pathological changes are initiated by vascular endothelial injury [36,37,38,39]. LDL-C can enter endothelial cells through endocytosis and thus accumulate at the lesion site [40]. Endothelial cell dysfunction is one of the main events in the physiological and pathological processes of AS [41].

The unstable/vulnerable plaques of AS (UA) patients are prone to rupture and thrombus formation, which are relevant to ischemic cerebral infarction caused by AS [42]. Wen et al. conducted a study in which AS patients were placed into stable plaque atherosclerosis (SA) and UA groups [43]. Patients with lipid-rich and necrotic plaques were diagnosed with UA, and the other patients were diagnosed with SA [44,45]. Some circRNAs can affect plaque stability by regulating the levels of inflammation and apoptosis in endothelial cells. Wen et al. found that circ_0006869 levels in the serum of UA patients were positively correlated with C-reactive protein (CRP) levels, and circ_0006896 can regulate STAT3 phosphorylation levels via the circ_0006896-miR-1264-DNMT1 axis in oxidized low-density lipoprotein (ox-LDL)-treated human umbilical vein endothelial cells (HUVECs) [43]. Abnormal STAT3 phosphorylation levels can cause the abnormal expression of adhesion molecules [46]; moreover, there is evidence that adhesion molecules can induce inflammation [47]. Treating cells with ox-LDL in vitro to mimic the pathological features of AS is widely recognized worldwide [48,49]. One study showed that circ_0006869 can influence inflammation through the circ_0006896-miR-1264-DNMT1 axis, thus affecting the course of AS and, ultimately, plaque stability. In addition, Yu et al. found that circ_0030042 could inhibit FOXO1 and beclin1 expression by regulating eIF4A3 nuclear translocation and obstructing eIF4A3 recruitment to mRNAs, thereby increasing aortic plaque stability in Apo-E^−/−^ mice [50].

One of the features of AS is low-grade and chronic inflammation in the arterial wall [51,52,53]. Some circRNAs have been shown to influence this process. Circ_RSF1 inhibits inflammation and apoptosis in ox-LDL-treated HUVECs by sponging miR-135b-5p to upregulate HDCA1, thereby inhibiting the progression of AS [33]. In addition, Yang et al. found that circ_0001445 was significantly decreased in ox-LDL-treated human aortic endothelial cells (HAECs) and serum from coronary artery disease (CAD) patients [54]. Circ_0001445 could inhibit ox-LDL-induced inflammation by attenuating the secretion of the pro-inflammatory factors TNF-α and IL-6 through the circ_0001445/miR-208p-5p/ABCG1 axis [54]. Lei et al. found that silencing circ_0090231 in ox-LDL-treated HUVECs could cause a decrease in the downstream expression of TXNIP due to miR-9-5p sponging, thus causing a decrease in the expression of inflammatory factors such as TNF- α, IL-1β, IL-6 [55]. In addition, the decrease in TXNIP downregulated the pro-apoptotic protein Bax and upregulated the anti-apoptotic protein BCL-2 in HUVECs [55], ultimately inhibiting apoptosis in these cells. Silencing circ_0090231 not only attenuates cellular inflammation and apoptosis levels in cellular models, but also lipid levels in ApoE^−/−^ mice [55,56]. Li et al. found that circ_CHMP5 regulates ROCK2 expression levels in HUVECs through the sponge miR-532-5p [56]. A related study showed that inhibition of the ROCK family, including ROCK2, could slow down the course of AS [57,58]. The scientists found that inhibiting circ_CHMP5 expression in vitro increased miR-532-5p expression, which caused a decrease in ROCK2 expression and ultimately a decrease in apoptosis and inflammatory indicators, such as BCL-2, Bax, IL-6, and IL-1β, as mentioned previously [56]. Ultimately, circ_CHMP5 knockdown could inhibit inflammation and apoptosis in HUVECs. Previously, it was found that circ_0003575 could affect apoptosis in aortic endothelial cells from B6 ApoE^−/−^ mice through the miR-148a-3p/FOXO3/FOXO4 axis [59]. It has been found that FOXO4 also affects endothelial cell apoptosis through the circ_0003575/miR-148a-3p/FOXO3/FOXO4 axis and may be able to inhibit AS by suppressing ROS and inflammatory cytokines from bone marrow-derived cells [60].

In conclusion, because they are a crucial step in AS injury, EC lesions play an important role in this disease. CircRNAs can affect the expression of downstream factors by acting on the corresponding microRNA sponges, ultimately changing the levels of inflammation and apoptosis in ECs and thus affecting the course of AS.

### 2.2. Foam Cells Formation and Death

A key step in the formation of fatty streaks is the formation and accumulation of foam cells. Modified low-density lipoprotein (LDL) internalization by macrophages leads to the formation of fatty streaks, the hallmark of early AS [61]. Macrophages can take up Ox-LDL via scavenger receptors on their surface thereby transforming into foam cells [62]. Vascular smooth muscle cells (SMC) can likewise express scavenger receptors and become foam cells [9]. Recent studies have suggested that approximately 50% of foam cells in human AS lesions [7] and 70% of those in mice [63] are derived from SMCs. Foam cell accumulation in the artery wall causes lipid plaque formation [64]. Foam cells die due to apoptosis or some other reason [40,65]. This foam cell death causes the appearance of lipid-rich necrotic nuclei in the center of the atherosclerotic plaque [40].

Wang et al. found that circ_0004104 overexpression in the human monocytic cell line (THP-1-derived macrophages) upregulates proatherosclerotic genes, such as IDO1, MMP-8, and CD40, and downregulates antiatherosclerotic genes, such as Apo A and RNASE1 [66]. Wang et al. found that circRNA transmembrane 7 superfamily member 3 (circ_TM7SF3) was notably higher in AS patients than healthy volunteers [67]. Furthermore, circ_TM7SF3 can target the miR-206/aspartyl (asparaginyl) β-hydroxylase (ASPH) axis to regulate inflammation and oxidative stress in AS cells in vitro [67]. In fact, silencing circ_TM7FS3 upregulates miR-206 and downregulates ASPH, in addition to alleviating ox-LDL injury, in both human monocyte-derived macrophages (hMDMs) extracted from the peripheral blood of volunteers and THP-1-derived macrophages [67]. He et al. found that circRNA sterol regulatory element binding transcription factor chaperone (circ_SCAP) was significantly elevated in atherosclerotic patients and THP-1 cells [68]. Circ_SCAP can promote phosphodiesterase 3B (PDE3B) expression by targeting miR-221-5p in THP-1 cells in vitro [68]. The circ_SCAP/miR-221-5p/PDE3B axis can regulate the nuclear factor kappa B (NF-κB) signaling pathway, which plays a critical role in inflammation [68,69,70].

In addition to common pathways such as inflammation and apoptosis that cause macrophage-derived foam cell death, pyroptosis is also a type of regulated cell death [65]. In 2001, Cookson et al. first used “pyroptosis” to describe the Caspase-1-dependent cell death process found in macrophages [71]. In pyroptosis, Caspase-1 activates the assembly of downstream inflammatory corpuscles, which is executed by gasdermin D of the gasdermin family [65]. This process is characterized by cell membrane rupture, pyroptosomal formation, and inflammatory factor release, leading to cell death [72]. Many studies have confirmed that macrophages are crucial to the process of AS and promote inflammation and plaque formation and rupture [73,74,75,76]. In the past, ox-LDL-induced macrophage death was considered to be apoptosis, but it was revealed that this process did not depend on caspase-3, the key executor of apoptosis [77]. Current research has revealed that pyroptosis may be the mechanism of ox-LDL-induced macrophage death [73,78,79]. Ge et al. found circ_0090231 overexpression in HAECs in vitro, and this molecule regulated cell injury and pyroptosis via the miR-635/NLRP3 axis [34]. Overexpression of NLPR3 can induce cleaved-caspase 1 protein expression and increase gasdermin N, the key protein of pyroptosis [34]. The authors concluded that circ_0090231 knockdown reduces pyroptosis and cell injury and improves cell viability in vitro [34]. In contrast, Guo M et al. found that circ_0029589 could reduce caspase-1 and gasdermin-N activity in macrophages in vitro [80]. In addition, circ_0029589 knockdown promoted the expression of pyroptosis-related genes [80] and inhibited AS through upregulating miR-424-5p to decrease insulin-like growth factor 2 (IGF2) in SMCs in vitro [81]. This research all indicates that circRNAs can influence the process of AS by regulating cell pyroptosis.

In summary, unlike their role in Ecs and SMCs, circRNAs affect the development of AS by regulating pyroptosis in macrophages, in addition to inflammatory pathways.

### 2.3. Fibrous Cap Formation and Atheroma Formation

In the case of severe vascular damage caused by some cytokines and growth factors (e.g., interleukin 1 and TNF), SMCs migrate to the luminal side of the vessel wall, thereby forming a fibrous cap [40]. SMCs, macrophages, and lymphocytes constitute a mature atheroma formation that expands into the lumen of the vessel and obstructs blood flow [62]. Fibrous cap formation is largely dependent on the migration and proliferation of SMCs, so this is another way in which SMCs influence the course of AS [41].

A previous study demonstrated that platelet-derived growth factors (PDGFs), proteolytic agents, and extracellular matrix proteins can induce SMC proliferation and migration from the media towards the intima during new vessel growth and atherogenesis and following vascular injury [82,83]. SMC migration from the media to the intima is an essential part of AS [84]. During migration, SMCs undergo phenotype switching and participate in atherosclerotic plaque development [85,86,87]. At the early stages of atherosclerotic plaques, SMCs transform to the macrophage-like phenotype in response to lipid deposition. These macrophage-like SMCs can phagocytize LDL and ox-LDL and contribute to the formation of foam cells [88]. However, there is an additional SMC phenotype that also plays an important role in the progression of AS. The contractile phenotype is typical of SMCs in healthy arteries; these SMCs have a very low proliferation rate and express proteins responsible for the physical contraction of cells [85,89]. As mentioned above, when SMC migration is induced by inflammatory factors such as PDGF, SMCs dedifferentiate to the synthetic phenotype and express pro-inflammatory factors that can be detected as macrophage markers [90,91,92,93]. These VSMC-derived macrophage-like cells promote AS due to their reduced ability to clear lipoproteins, dying cells, and necrotic debris and by exacerbating inflammation [88]. In summary, SMC migration, proliferation, and phenotype switching can influence AS development by affecting inflammation levels in SMCs. Chen et al. found that circ_WDR77 can sponge miR-124, which can regulate the expression of fibroblast growth factor 2 (FGF2) [94]. Silencing circ_WDR77 can inhibit the amount and distance of SMC migration in vitro via the circ_WDR77-miR-124-FGF2 pathway [94]. Peng et al. found that downregulating circ_DCHR24 also inhibited in vitro ox-LDL-induced conversion of human aortic vascular smooth muscle cells (HA-VSMCs) to the contractile phenotype via the miR-149-5p/MMP9 axis [95]. CircRNA ubiquitin protein ligase E3 component-recognin 4 (circ_UBR4) was shown to be upregulated in both ox-LDL-treated SMCs and serum from AS patients [96,97]. Circ_UBR4 has many downstream targets, such as miR-637, miR-185-5p, and miR-491, and its knockdown can reduce SMC migration and proliferation induced by ox-LDL [96,97,98]. As a multi-target circRNA, circ_UBR4 may thus be a therapeutic target for AS.

Kruppel-like factor 5 (KLF5) is a member of the KLF family, a group of DNA-binding transcriptional regulators, and is thought to be a protective factor in AS [99,100,101]. KLF5 has many targets, including PDGF-A, cyclin D1, cyclin B, Egr-1, VEGF, and PAI-1, and promotes SMC proliferation, migration, apoptosis, and vascular inflammation through them [102,103,104,105,106,107,108,109]. A study by Zhao et al. showed that knocking down circ_USP36 could increase miR-182-5p and subsequently reduce KLF5 expression to promote Bax expression [110]. Moreover, downregulating circ_USP36 promoted apoptosis in ox-LDL-treated human umbilical vein smooth muscle cells (HUVSMCs) while also attenuating ox-LDL-induced injury [110]. Sun et al. found that circ_RUSC2 can promote the expression of miR-661 target gene SYK in human coronary artery smooth muscle cells (HCASMCs) [111]. In addition, SKY upregulation inhibited apoptosis in HCASMCs [111]. The scientists also found that circ_RUSC2 could inhibit the expression of SM22α, a marker of contractile VSMCs; this finding also implies that circ_RUSC2 inhibits the formation of contractile SMCs [111].

The cell cycle can be divided into interphase, includes G1, S, and G2, and the mitotic (M) phase, which includes prophase, metaphase, anaphase, and telophase. Zhang et al. found that circ_PTPRA can regulate the cell cycle to affect AS via the circ_PTPRA/miR-636/SP1 axis [112]. Circ_PTPRA inhibition suppressed cell growth by improving G0/G1 phase cell distribution and reducing S phase cell distribution; miR-636 inhibition reversed this effect [112]. SP1 is a transcription factor that promotes cell proliferation and regulates AS progression [112,113,114]. SP1 inhibition can improve cell distribution in the G0/G1 phase, suppress the levels of cyclins E and D (cell proliferation-related proteins), and promote the levels of Bax, Bad, and cleaved caspase 3 (apoptosis markers); as such, SP1 inhibition can reduce cell proliferation and promote cell apoptosis in VSMCs in vitro [112]. Deniaud et al. also found that SP1 overexpression could cause an increase in apoptosis [115]. Similarly, Lin et al. found that circ_0044073 was upregulated in AS patients and could bind with miR-107 [116]. Furthermore, circ_0044073 overexpression improved the ratio of HUVECs and HUVSMCs in G2/M and S phase, indicating that circ_0044073 overexpression can promote cell proliferation and invasion [116]. miR-107 overexpression reverses the effects of circ_0044073 and inhibits the expression of JAK1, which is a key factor in many inflammatory diseases [116,117].

In conclusion, circRNA can influence the course of AS by altering SMC proliferation, migration, phenotype transition, and cell cycle distribution.

There are also many studies on the effects of CircRNA on AS, which I have listed in the Table 1 due to their more repetitive mechanisms of action.

## 3. Special CircRNAs

### 3.1. Circ_ANRIL

ANRIL (antisense non-coding RNA in the INK4 locus), or CDKN2B-AS1, is located at the human CDKN2A/B locus at 9p21.3 and is transcribed in the antisense direction of the INK4b-ARF-INK4a gene cluster [119,120,121]. Previous work has indicated that ANRIL is capable of forming RNA circles [19]. In addition, circ_ANRIL RNA levels are higher than the levels of linear ANRIL (linANRIL) in different human cell types and tissues [122]. Circ_ANRIL was also more stable than linANRIL [17,18,122]. Ox-LDL treatment significantly increased ANRIL expression in HUVECs and HA-VSMCs [123]. Similarly, serum ANRIL levels were significantly higher in AS patients than healthy subjects [123]. Circ_ANRIL affects AS by modulating vascular endothelial cells (VECs), vascular smooth muscle cells (VSMCs), inflammatory responses, and cell apoptosis and proliferation. Furthermore, Holdt et al. found that in rats with low circ_ANRIL expression, ECs were closely arranged and spindle-shaped, and all organelles had a normal structure without denaturation [124]. However, in the circ_ANRIL overexpression group, the arterial structure was more disorganized, more thrombi appeared, ECs were less abundant, and the number of physalides observed in the cytoplasm and mitochondrial welling were increased [124]. This disorganization contributes to an irregular blood flow that causes unfavorable vascular responses, eventually leading to vascular diseases [125,126]. Overall, circ_ANRIL overexpression can increases the risk of AS by changing the vessel structure. ANRIL can mediate AS through the TNF-α-NF-κB-ANRIL/YY1-IL6/8 pathway [127]. ANRIL knockdown decreased NF-κB mRNA levels and the expression of IL6 and other pro-inflammatory cytokines [127,128]. In a study from Gareus R et al., blocking the endothelial NF-κB signaling pathway had obvious protective effects on AS, and DNIκBα was shown to block the nuclear translocation of NF-κB [129]. DNIκBα overexpression nearly completely inhibited atherosclerotic plaque development in ApoE^−/−^ mice [129]. Dehydroxymethylepoxyquinomicin (DHMEQ) has been shown to inhibit the NF-κB pathway [130], potentially offering a new type of treatment. Caspase recruitment domain 8 (CARD8; also known as TUCAN) comprised a C-terminal CARD domain and an N-terminal FIIND (domain with function to find) domain [131]. Bai et al. found that CARD8 was significantly reduced by 48% in HUVECs transfected with ANRIL-specific siRNA compared with cells transfected with NC siRNA [132]. These data suggest that ANRIL regulates the expression of CARD8. Paramel et al. knocked down CARD8 in HUVECs, and the inflammatory cytokines CXCL1, MCP-1, and IL-6 were significantly reduced in both the lysates and culture medium [133]. In AS, a variety of inflammatory cells and inflammatory factors play significant roles throughout its pathogenesis [134]. In this manner, CADR8 can regulate the expression of inflammatory factors to influence the progression of AS.

**Figure 1:**
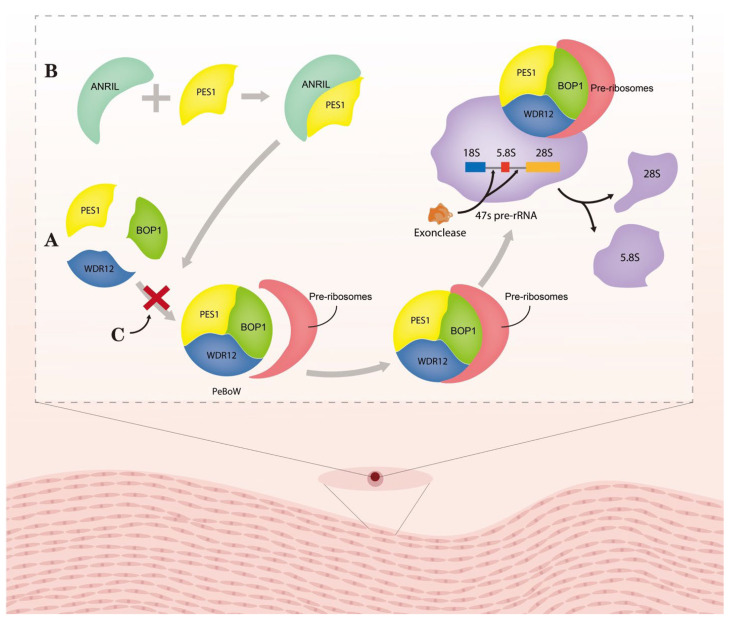
Molecular mechanism of circ_ANRIL controlling PeBoW complex fuction. (**A**) PeBoW complex assembles with the pre-ribosome and binds the precursor rRNA (pre-rRNA), thereby facilitating the processing of 47S pre-rRNA into mature 28S and 5.8S rRNAs by nucleic acid exonucleases. (**B**) Lesca et al. found a strong interaction between circRNA and PES1 by RNA immunoprecipitation (RIP). (**C**) Circ_ANRIL binding to the complex of PES1 inhibited the formation of PeBoW complex, thus suppressing the formation of 28S and 5.8S rRNAs. Abbreviation: ANRIL: circ_ANRIL; PES1: pescadillo homologue1; BOP1: block of proliferation 1; and WDR12: WD-repeat protein 12.

Gomez et al. found that during ox-LDL-induced plaque formation, SMCs switch their phenotype from contractile to synthetic, which is commonly identified by proliferation, migration, and ROS production [85]. ANRIL overexpression could induce cell growth and ROS production in HA-VSMCs, whereas siRNA-mediated ANRIL silencing blocked the cell growth and ROS activation induced by ox-LDL treatment [135]. Similarly, ANRIL silencing markedly reduced ox-LDL-induced HASMC migration [135]. OPN collagen type III, cyclophilin 1, and α-SMA are synthetic phenotype markers of VSMCs; ANRIL induced OPN collagen type III expression, but its silencing decreased cyclophilin 1 and α-SMA levels in HASMCs [135]. Previous studies found that histone deacetylase HDAC3 interacts with histone methylation-associated protein WDR5 to recruit other histone-modifying complexes to regulate the chromatin structure and the transcription of genes involved in invasion/migration activity in vitro [136]. NADPH oxidase (NOX) was recently reported as associated with altered SMC phenotypes [137]. ANRIL silencing could disrupt the interaction between endogenous WDR5 and HDAC3 proteins and cause a significant NOX1 loss at the mRNA and protein levels [135]. Enhanced cell proliferation, ROS production, and migration after ox-LDL treatment were all attenuated significantly by siRNA-mediated NOX1 knockdown [135,137]. Pescadillo homologue 1 (PES1) is a component of the PES1–BOP1–WDR12 (PeBoW) complex [138]. A PeBoW homologue complex consisting of Nop7 (Yph1p), Erb1 and Ytm1p was found in yeast [139]. Mutants of Nop7 and Ytm1 could inhibit rRNA processing and cell cycle progression [139]. In addition, circ_ANRIL acts a molecular inhibitor of PES1 by binding to the C-terminal domain of PES1 to inhibit ribosome maturation (Figure 1) [122]. Lecca et al. found that circ_ANRIL overexpression inhibits cell proliferation, as well as promoting apoptosis, in VSMCs [122]. Ultimately, circ_ANRIL acts as a molecular inhibitor of PES1 and prevents ribosome maturation by binding to its C-terminal structural domain, thus regulating cell proliferation and apoptosis [122]. In this manner, circ_ANRIL induces apoptosis and decreases cell proliferation that can stop the progression of AS [122].

### 3.2. Circ_CHFR

**Figure 2:**
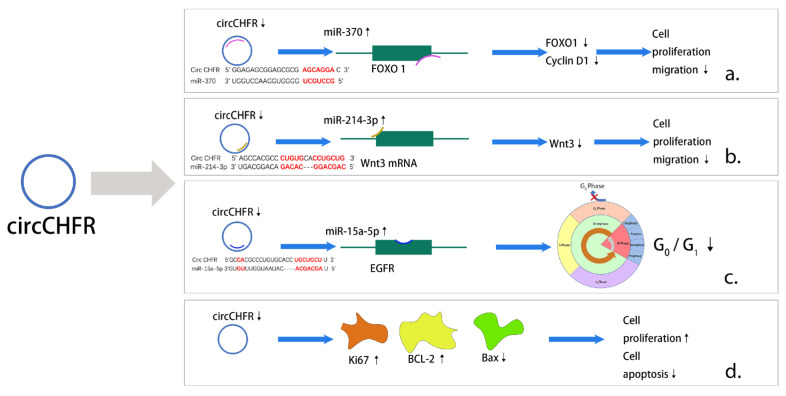
Major pathways of action of circ_CHFR in AS. (**a**) Downregulation of circ_CHFR inhibits the expression of FOXO1 as well as Cyclin D1 through upregulating activity of miR-370 ultimately causing a reduction in cell proliferation and migration. (**b**) Downregulation of circ_CHFR inhibits Wnt3 expression through upregulating activity of miR-214-3p ultimately causing a decrease in cell proliferation and migration. (**c**) Downregulation of circ_CHFR by upregulating activity of miR-15a-5p binds to EGFR and ultimately causes a decrease in the G0/G1 ratio in the cell cycle. (**d**) Unlike previous studies, downregulation of circ_CHFR promotes cell proliferation and, in addition, causes a decrease in apoptosis.

Circ_CHFR (checkpoint with forkhead-associated and ring-finger domains) is a circRNA marked by the CHFR gene, which is involved in VEMC and EC proliferation and migration [140,141]. Circ_CHFR was significantly increased in serum from patients with AS and several types of in vitro cells, such as VSMCs, ECs, and human brain microvessel endothelial cells (HBMECs) [141,142,143]. There are several pathways and mechanisms that contribute to AS, including the miR-214-3p/Wnt3/β-catenin and miR-370/FOXO1/Cyclin D1 axes [140,142]. Cell cycle arrest occurred at the G0/G1 phase upon ox-LDL treatment in HBMECs, but a circ_CHFR inhibitor attenuated this effect mediated by miR15a-5p [141]. An analysis of circ_CHFR and some miRNA sequences suggested that there were several complementary binding sites within circ_CHFR and miRNAs [140,143,144], indicating that circ_CHFR can sponge miRNA [142]. Relatedly, Zhuang et al. found serum miR-214-3p levels to be significantly lower in AS patients than normal subjects [142]. In an in vitro study, Yang et al. found that miR-370 expression in VSMCs was downregulated by circ_CHFR knockdown [140]. Additionally, miRNAs can regulate downstream molecules to affect cell proliferation, migration, and invasion and inflammatory factor expression. MiR-15a-5p can bind with epidermal growth factor receptor (EGFR) to promote proliferation and inhibit apoptosis in HBMECs [141], and miR-214-3p overexpression inhibits Wnt3 mRNA and protein expression to attenuate ox-LDL-induced injuries in VSMCs [142]. Recent data suggest that ox-LDL could inhibit cell proliferation in vitro [141]; markers of cell proliferation, including the proliferation-related protein Ki67, were downregulated, the pro-apoptotic protein Bax was upregulated, and the anti-apoptotic protein BCL-2 was downregulated [141,143,144]. Circ_CHFR silencing reversed the above effects to promote proliferation [141,143,144]. In summary, circ_CHFR can regulate the cell cycle, proliferation, migration, and apoptosis through multiple downstream pathways, ultimately affecting the progression of AS (Figure 2).

### 3.3. Circ_USP

CircRNA ubiquitin-specific protease 36 (circ_USP36, also known as circ_0003204) expression was shown to be upregulated in ox-LDL-treated HAECs and HUVECs [145,146,147,148]. Zhang et al. showed that circ_USP36 expression is significantly higher in plasma from AS patients than non-AS patients [146]. Circ_USP36 can influence the viability and migration of HAECs in vitro by regulating WNT4 and sponging miR-637 [145]. The sponge activity of miRNA is the most commonly described function of circRNA; in this manner, circRNAs can reduce the expression complexity of target genes or limit miRNA translation by blocking RNA-induced silencing [26,149,150]. Huang et al. found that enhanced miR-637 expression can lead to decreased levels of WNT4 and vice versa [145]. Overexpression of circ_USP36 led to decreased EC migration and viability to promote AS development, and knocking out WNT4 attenuated this inhibition [145]. In addition, circ_USP36 can decrease HAEC viability, migration, and capillary formation via the miR-370/TGFβR2/phosph-SMAD3 axis [146]. Circ_USP36 can sponge miR-370 to reduce its expression, and miR-370 upregulates TGFβR2 and phosph-SMAD3, which can block HAEC proliferation, migration, and capillary formation to exacerbate the progression of AS [146]. Zhang et al. similarly found that knocking down circ_USP36 can protect ECs from ox-LDL-induced injury via the miR-491-5p-ICAM1 pathway [151]. Peng et al. showed that circ_USP36 can alter HUVEC damage caused by ox-LDL in vitro via the circ_USP36/miR-98-5p/VCAM1 (vascular cell adhesion molecule 1) axis [147]. Circ_USP36 can sponge miR-98-5p to suppress its expression, and miR-98-5p directly targets VCAM1 [147]. VCAM1 is an adhesion molecule that participates in AS, and some researchers have already found that it can regulate the initiation of AS [147,152]. Oxidative stress is an imbalance in favor of increased ROS generation and/or reductions in the innate anti-oxidant defense systems [153]. Nucleotide-binding oligomerization domain (Nod2), a member of the nucleotide-binding oligomerization domain-like receptor (NLR) family, is involved in the activation of NF-κB, MAPKs, and other pro-inflammatory molecules; it can also enhance the inflammatory response and regulate the body’s epidemic immunity response [154]. Zhang et al. revealed that downregulating circ_USP36 can ablate ox-LDL-induced oxidative stress and apoptosis in HEACs via miR-330-5p targeting Nod2 [155]. What is more, Zhou et al. found that circ_USP36 regulates ox-LDL-induced autophagic granulocyte death [156], and circ_USP36 was upregulated in ox-LDL-induced HUVSMCs [110]. This group also showed that circ_USP36 knockdown inhibits the proliferation, migration, and invasion of ox-LDL-treated HUVSMCs [110].

Circ_USP36 may act as not only an upstream regulator of miRNAs but also a downstream factor that inversely regulates miRNAs to affect the process of AS. Tang et al. found that the lncRNA ZFAS1 can affect AS through the miR-654-3p/ADAM10 and RAB22A axis by mediating the inflammatory response, as well as cholesterol metabolism [157]. ADAM10 and RAB22A have been shown to facilitate AS [157]. Circ_CORO1C was shown to have an interaction site with miR-654-3p; in addition, circ_USP7 is downstream of miR-654-3p, which is part of a pathway that regulates laryngeal squamous cell carcinoma progression [158]. These two studies confirm that circ_CORO1C can directly regulate miR-654-3p, which, in turn, may be able to mediate the interaction of miR-654-3p with ADAM10 and RAB22A in another pathway, thus affecting AS [157,158]. Circ_USP7, a downstream factor of miR-654-3p, can bind to miR-654-3p; this action may be able to reverse the expression or activity of miR-654-3p, thus affecting the interaction of miR-654-3p with ADAM10 in other pathways [157,158].

## 4. Clinical Applications of CircRNA

AS can cause many diseases, among which coronary heart disease and stroke seriously jeopardize human health [159,160]. Some scholars have now found the diagnostic and therapeutic value of circRNA in the clinic. For example, Han et al. found that patients with acute ischemic stroke (AIS) had significantly higher levels of circ_HECTD1 in plasma compared with controls [161], which is of clinical significance for the early prevention and diagnosis of AIS. Similarly, in a study by Yu et al., the expression level of circ_0030042 was significantly lower in coronary arteries containing atherosclerotic plaques than in coronary arteries without atherosclerotic plaques [50]. In addition, the expression level of circ_0030042 in peripheral mononuclear cells of patients with coronary artery disease was also lower than that of controls [50]. From the above, it can be seen that changes in the expression level of circRNA in plasma or peripheral blood cells have the potential to become a tool for clinical prediction and diagnosis of the disease. Other scholars have found that overexpression of circ_Hipk3 can induce myocardial regeneration and angiogenesis, which has certain significance for the treatment of myocardial infarction [162]. In addition, as previously mentioned, statins are an effective class of therapeutic agents for AS, and it does more than just inhibit HMG-CoA. Atorvastatin has been found to significantly reduce the expression level of circ_0004831 in ox-LDL-treated HUVECs [163]. Su et al. found that atorvastatin attenuates apoptosis, cell cycle arrest, oxidative stress, and inflammation by inhibiting the expression of circ_0004831, which is mediated through the circ_0004831/miR-182-5p/CXCL12 axis [163]. In summary, circRNA has the value of prediction, diagnosis, and treatment for coronary heart disease and stroke caused by AS.

## 5. Conclusions

As previously mentioned, AS is closely correlated with strokes, and circRNA can be a predictor of strokes, which could be of great significance for early treatment and prevention. Zu et al. found that serum circRNA levels were increased significantly in patients with large artery AS, and small artery occlusion and cardioembolism strokes were also significantly increased [164]. Additionally, Li et al. found that serum circ_0001599 levels were significantly higher in stroke patients than healthy controls [165]. Moreover, stroke severity (including infarct size and clinical symptoms) was positively correlated with the circ_0001599 level [165]. Therefore, serum circRNA levels in patients may be a reliable predictor of stroke in the future. Yang et al. observed a correlation between circ_LMF1 knockdown and proliferation and migration in HASMCs [166]. As mentioned before, various circRNAs that can affect the proliferation and migration of SMCs and ECs could possibly be used to treat AS. Some researchers believe that acute cerebral infarction is related mainly to the rupture of vulnerable carotid atherosclerotic plaques [167]. Hernan et al. revealed that in patients with carotid plaques, miR-221 levels were higher in asymptomatic patients than acute patients, but there was no significant difference in circ_248 [168]. Due to its potential miR-221 binding site, circ_284 is a possible miR-221 inhibitor, making it a candidate for standardizing miR-221 measurements, which could indirectly reflect carotid plaque stability and thus predict the occurrence and prognosis of ischemic events [168].

CircRNA has many features such as conserved sequence, tissue specificity, high stability, and high abundance, leading to its excellent suitability as a biomarker. In addition, based on the structural stability, low immunogenicity, and protein translation function of circRNAs, circRNAs represent a new platform for gene expression. Researchers can encode any protein of interest through appropriate sequence design and in vitro preparation. CircRNAs are a new type of “programmed drug” with unlimited potential to express any therapeutic protein in vivo, which may replace or augment the current pattern of drug use. In conclusion, circRNAs play very important roles in the process of AS and could be of great value for the prediction and treatment of AS.

## Figures and Tables

**Table 1:** Regulatory roles of circular RNAs(circRNAs) in atherosclerotic models.

CircRNA	Model		Expression Level in Model	Molecular Pathways	Biological Process	References
	Cell/Animal	Chemical				
Circ_0030042	HUVEC	Ox-LDL (100 ug/mL)	HUVEC ↓	Circ_0030042 inhibit eIF4A3 stimulate FOXO1 and beclin 1	Autophagy	[50]
Circ_HIPK3	HUVEC ApoE^−/−^ mice	Ox-LDL (100 ug/mL, 12 h) High-fat diet (78.85% basic feed, 0.15% cholesterol and 21% fat,16 weeks)	HUVEC ↓ ApoE^−/−^ mice ↓	Circ_HIPK3 inhibit miR-190b inhibit ATG7	Autophagy	[118]
Circ_RSF1	HUVEC	Ox-LDL (100 ug/mL, 48 h)	HUVEC ↓	Circ_RSF1 inhibit miR-135b-5p inhibit HDAC1	Cell proliferation Cell apoptosis	[33]
Circ_0001445	HAEC	Ox-LDL (100 ug/mL, 0–72 h)	HAEC ↓	Circ_0001445 inhibit miR-208b-5p inhibit ABCG1	Cell proliferation Cell migration inflammation	[54]
Circ_0090231	HUVEC	Ox-LDL (75 ug/mL, 48 h)	HUVEC↑	Circ_0090231 inhibit miR-9-5p inhibit TXNIP	Angiogenesis Oxidative stress Inflammation Apoptosis	[55]
Circ_0003575	HUVEC	Ox-LDL (100 ug/mL, 24 h)	HUVEC↑	Circ_0003575 inhibit miR-532-5p inhibit ROCK2	Cell proliferation Angiogenesis Cell apoptosis Inflammation	[56]
Circ_DHCR 24	HA-VSMC	PDGF-BB (2.5 mg/L, 24 h)	HA-VSMC↑	Circ_DHCR 24 inhibit miR-149-5p inhibit MMP9	Cell proliferation Cell migration Phenotypic switch	[95]
Circ_UBR4	HVSMC	Ox-LDL (100 ug/mL, 48 h)	HVSMC↑	Circ_UBR4 inhibit miR-637 inhibit FOXO4	Cell proliferation Cell migration	[96]
Circ _UBR4	VSMC	Ox-LDL (100 ug/mL, 48 h)	VSMC ↑	Circ_UBR4 inhibit miR-185-5p inhibit FRS2	Cell proliferation Cell migration	[97]
Circ_UBR4	Human VSMC	Ox-LDL (0, 25, 50, 100 ug/mL, 48 h)	Human VSMC ↑	Circ_UBR4 inhibit miR-491-5p inhibit NRP2	Cell proliferation Cell migration	[98]
Circ_PTPRA	VSMC	Ox-LDL (unknow)	VSMC ↑	Circ_PTPRA inhibit miR-636 inhibit SP1	Cell proliferation Apoptosis	[112]
Circ_0090231	HAEC	Ox-LDL (25 ug/mL, 24 h)	HAEC ↑	Circ_0090231 inhibit miR-635 inhibit NLP3	Cell pyrotosis	[34]
Circ_TM7SF3	THP-1-derived macrophage hMDMs	Ox-LDL (50 ug/mL, 24 h)	Macrophage ↑	Circ_TM7SF3 inhibit miR-206 inhibit ASPH	Cell apoptosis Inflammation Oxidative stress	[67]
Circ_SCAP	THP-1-derived macrophage	Ox-LDL (50 ug/mL, 24 h)	Macrophage ↑	Circ_SCAP inhibit miR-221-5p inhibit PDE3B	Cell proliferation Cell apoptosis Inflammation Oxidative stress	[68]

Abbreviation: EC: endothelial cells; HAEC: human aortic endothelial cell; HA-VSMC: human aortic vascular smooth muscle cell; hMDMs: human monocyte-derived macrophages; HUVEC: human umbilical vein endothelial cell; HVSMC: human vascular smooth muscle cell; ox-LDL: oxidized low-density lipoprotein; and PDGF: platelet-derived growth factor. ↓: It represents the diminished activity of this CircRNA in this cell. ↑: Itrepresents the enhanced activity of this CircRNA in this cell.

## Data Availability

Data sharing is not applicable to this article as no datasets were generated or analyzed during the current study.

## Abbreviations

ANRIL	antisense non-coding RNA in the INK4 locus
Apo-	apolipoprotein
AS	atherosclerosis
ASPH	aspartyl (asparaginyl) β-hydroxylase
CAD	coronary artery disease
CHD	coronary heart disease
Circ_CHFR	the checkpoint with forkhead-associated and ring-finger domains
CircRNAs	circular RNAs
Circ_SCAP	circular RNA sterol regulatory element binding transcription factor chaperone
Circ_TM7SF3	circular RNA transmembrane 7 superfamily member 3
Circ_USP36	circular RNA ubiquitin-specific protease 36
CRP	C-reactive protein
DHMEQ	dehydroxymethylepoxyquinomicin
EC	endothelial cells
EGFR	epidermal growth factor receptor
FGF2	fibroblast growth factor 2
HAEC	human aortic endothelial cell
HA-VSMC	human aortic vascular smooth muscle cell
HCASMC	human coronary artery smooth muscle cell
hMDM	human monocyte-derived macrophage
HUVEC	human umbilical vein endothelial cell
HUVSMC	human umbilical vein smooth muscle cell
HVSMC	human vascular smooth muscle cell
IGF2	insulin-like growth factor 2
KLF5	kruppel-like factor 5
NF-κB	nuclear factor kappa B
NLR	nucleotide binding oligomerization domain-like receptor
Nod2	nucleotide-binding oligomerization domain
NOX	NADPH oxidases
ox-LDL	oxidized low-density lipoprotein
PDE3B	phosphodiesterase 3B
PeBoW	PES1–BOP1–WDR12
PES1	pescadillo homologue 1
SA	stable plaque atherosclerosis
SMC	smooth muscle cell
UA	unstable/vulnerable plaque atherosclerosis
VCAM1	vascular cell adhesion molecule 1
VECs	vascular endothelial cells
